# Supplementary figures and images for: MicroRNA biogenesis pathway genes polymorphisms and cancer risk: a systematic review and meta-analysis
Source: PeerJ. 2016 Dec 7;4:e2706. doi: 10.7717/peerj.2706 (PMC5147022; doi:10.7717/peerj.2706)

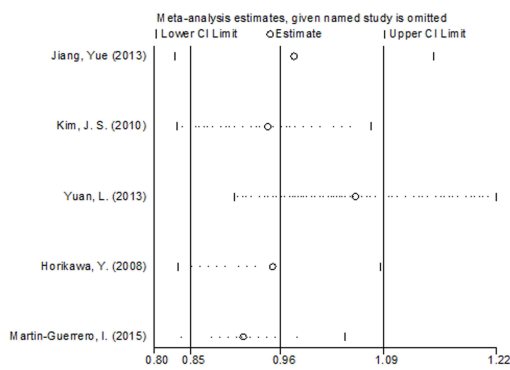

A:rs10719T>C

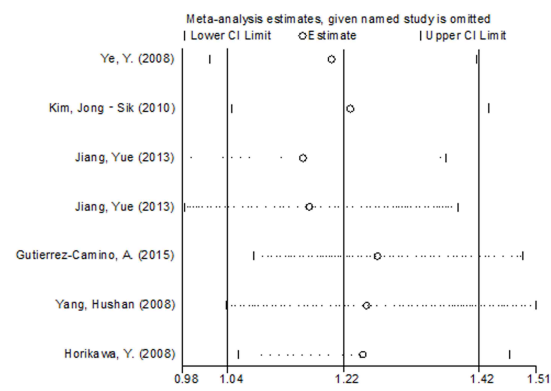

B:rs417309G>A

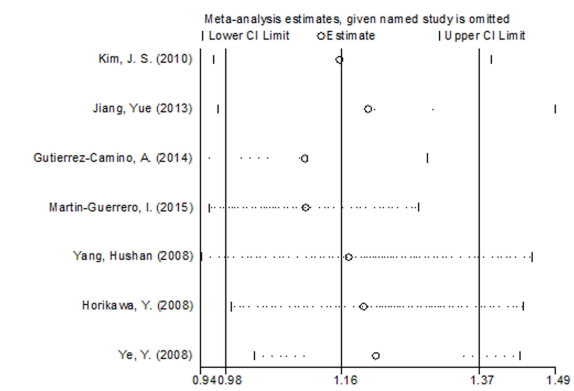

C: rs1640299T>G

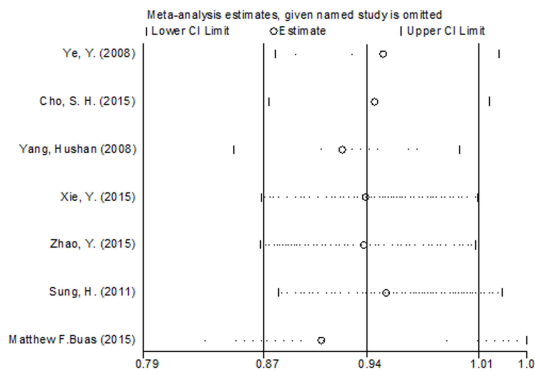

D:rs11077A>G

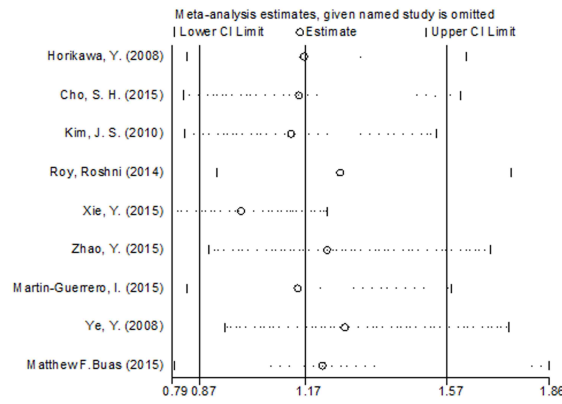

E:rs14035C>T

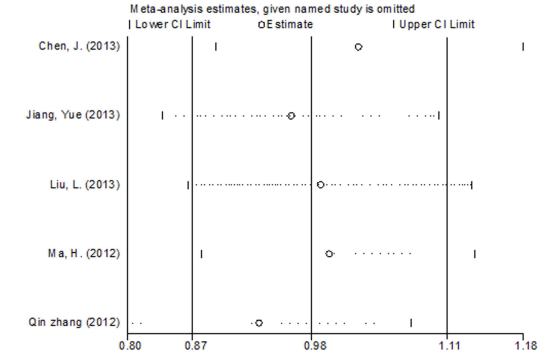

F: rs3803012A>G

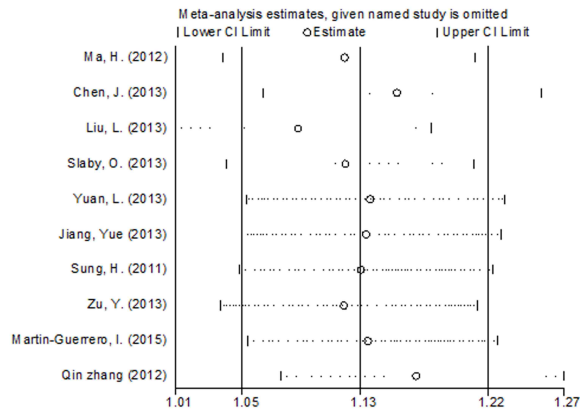

G:rs1057035T>C

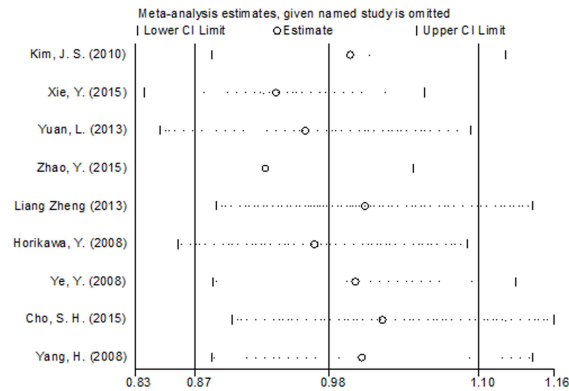

H:rs3742330A>G

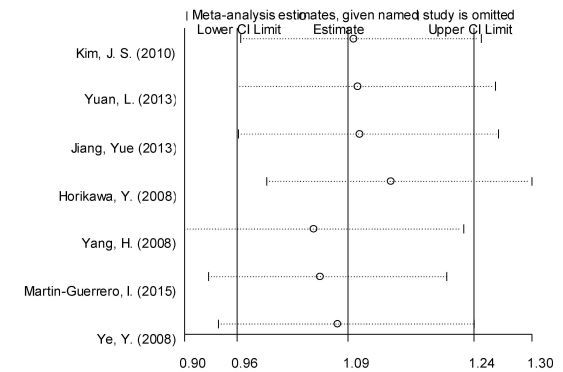

I:rs13078T>A

Supplement: Figure S1 [file peerj-04-2706-s002.pdf]
