# Supplementary material for: MicroRNA biogenesis pathway genes polymorphisms and cancer risk: a systematic review and meta-analysis
Source: PeerJ. 2016 Dec 7;4:e2706. doi: 10.7717/peerj.2706 (PMC5147022; doi:10.7717/peerj.2706)
Supplement: Table S1 — Characteristics of eligible studies for each SNP in the meta-analysis [file peerj-04-2706-s003.docx]

**Table S1． Characteristics of eligible studies for each SNP in the meta-analysis**

| **gene** | **reference** | **Year** | **Cancer type** | **Country** | **Ethnicity** | **controls** | **nos** | **Genotype-case** | | | **Genotype-control** | | | **method** | **HWE *P*-value** |
| --- | --- | --- | --- | --- | --- | --- | --- | --- | --- | --- | --- | --- | --- | --- | --- |
| ***DROSHA*** | rs10719T>C |  |  |  |  |  |  | TT | TC | CC | TT | TC | CC |  |  |
|  | Jiang, Y | 2013 | breast cancer | China | Asian | HB | 7 | 433 | 346 | 68 | 463 | 353 | 62 | TaqMan | 0.63 |
|  | Kim, J. S. | 2010 | lung cancr | Korea | Asian | HB | 7 | 59 | 29 | 9 | 52 | 38 | 7 |  | 0.99 |
|  | Yuan, L. | 2013 | bladder cancer | China | Asian | HB | 7 | 352 | 278 | 54 | 413 | 275 | 39 | TaqMan | 0.44 |
|  | Horikawa, Y. | 2008 | renal cell carcinoma | American | Caucasian | PB | 8 | 161 | 75 | 16 | 155 | 76 | 15 | SNPlex | 0.18 |
|  | Martin-Guerrero, I. | 2015 | Lymphocytic Leukemia | Spanish | Caucasian | HB | 7 | 71 | 26 | 5 | 193 | 134 | 18 | Taqman | 0.39 |
| ***DGCR8*** | rs417309G>A |  |  |  |  |  |  | GG | GA | AA | GG | GA | AA |  |  |
|  | Ye, Y. | 2008 | esophageal cancer | American | Caucasian | HB | 7 | 286 | 57 | 3 | 299 | 45 | 2 | SNPlex | 0.83 |
|  | Kim, Jong‐Sik | 2010 | lung cancr | Korea | Asian | HB | 7 | 90 | 8 | 0 | 88 | 9 | 0 | TaqMan | 0.63 |
|  | Jiang, Y | 2013 | breast cancer | China | Asian | HB | 7 | 830 | 68 | 3 | 910 | 49 | 1 | TaqMan | 0.69 |
|  | Jiang, Y | 2013 | breast cancer | China | Asian | HB | 7 | 771 | 89 | 0 | 826 | 67 | 0 | TaqMan | 0.24 |
|  | Gutierrez-Camino, A. | 2015 | Lymphocytic Leukemia | Spanish | Caucasian | HB | 7 | 98 | 6 | 1 | 302 | 41 | 3 | SNaPshot | 0.23 |
|  | Yang, Hushan | 2008 | bladder cancer | China | Caucasian | HB | 7 | 620 | 109 | 11 | 631 | 102 | 5 |  | 0.69 |
|  | Horikawa, Y. | 2008 | renal cell carcinoma | American | Caucasian | PB | 8 | 243 | 30 | 4 | 243 | 34 | 1 | SNPlex | 0.87 |
| ***DGCR8*** | rs1640299T>G |  |  |  |  |  |  | TT | TG | GG | TT | TG | GG |  |  |
|  | Kim, J. S. | 2010 | lung cancr | Korea | Asian | HB | 7 | 58 | 33 | 7 | 52 | 40 | 5 |  | 0.44 |
|  | Jiang, Y | 2013 | breast cancer | China | Asian | HB | 7 | 465 | 330 | 54 | 476 | 357 | 58 | TaqMan | 0.41 |
|  | Gutierrez-Camino, A. | 2014 | Lymphocytic Leukemia | Spanish | Caucasian | HB | 7 | 73 | 95 | 31 | 93 | 174 | 82 |  | 0.97 |
|  | Martin-Guerrero, I. | 2015 | Lymphocytic Leukemia | Spanish | Caucasian | HB | 7 | 38 | 43 | 22 | 85 | 182 | 82 | Taqman | 0.42 |
|  | Yang, Hushan | 2008 | bladder cancer | China | Caucasian | HB | 7 | 226 | 354 | 157 | 203 | 363 | 171 |  | 0.72 |
|  | Horikawa, Y. | 2008 | renal cell carcinoma | American | Caucasian | PB | 8 | 65 | 151 | 61 | 67 | 136 | 75 |  | 0.73 |
|  | Ye, Y. | 2008 | esophageal cancer | American | Caucasian | HB | 7 | 87 | 173 | 87 | 97 | 172 | 76 | SNPlex | 0.99 |

**Table S1 (continued)**

| **gene** | **reference** | **Year** | **Cancer type** | **Country** | **Ethnicity** | **controls** | **nos** | **Genotype-case** | | | **Genotype-control** | | | **method** | **HWE *P*-value** |
| --- | --- | --- | --- | --- | --- | --- | --- | --- | --- | --- | --- | --- | --- | --- | --- |
| ***XOP5*** | rs11077A>G |  |  |  |  |  |  | AA | AG | GG | AA | AG | GG |  |  |
|  | Ye, Y. | 2008 | esophageal cancer | American | Caucasian | HB | 7 | 101 | 146 | 53 | 118 | 137 | 40 | SNPlex | 0.98 |
|  | Cho, S. H. | 2015 | Colorectal Cancer | Korean | Asian | HB |  | 333 | 74 | 1 | 337 | 61 | 2 | PCR-RFLP | 0.67 |
|  | Yang, Hushan | 2008 | bladder cancer | China | Caucasian | HB | 7 | 248 | 356 | 114 | 241 | 363 | 122 |  | 0.46 |
|  | Xie, Y. | 2015 | gastric cancer | China | Asian | HB | 7 | 119 | 17 | 1 | 123 | 18 | 1 |  | 0.70 |
|  | Zhao, Y. | 2015 | colorectal cancer | China | Asian | HB | 7 | 143 | 19 | 1 | 123 | 18 | 1 |  | 0.70 |
|  | Sung, H. | 2011 | breast cancer | Korea | Asian | HB | 7 | 473 | 82 | 4 | 501 | 64 | 2 | TaqMan | 0.98 |
|  | Matthew F.Buas | 2015 | esophageal cancer | - | Caucasian | HB | 7 | 1909 | 2826 | 1045 | 1097 | 1557 | 552 |  | 0.99 |
| ***RAN*** | rs14035C>T |  |  |  |  |  |  | **CC** | **CT** | **TT** | **CC** | **CT** | **TT** |  |  |
|  | Horikawa, Y. | 2008 | renal cell carcinoma | American | Caucasian | PB | 8 | 143 | 110 | 23 | 129 | 125 | 24 | SNPlex | 0.41 |
|  | Cho, S. H. | 2015 | Colorectal Cancer | Korean | Asian | HB | 7 | 267 | 128 | 13 | 233 | 150 | 17 | PCR-RFLP | 0.24 |
|  | Kim, J. S. | 2010 | lung cancer | Korea | Asian | HB | 7 | 65 | 23 | 5 | 52 | 33 | 5 |  | 0.94 |
|  | Roy, Roshni | 2014 | oral cancer | India | Asian | HB | 7 | 258 | 157 | 24 | 300 | 125 | 13 | Taqman | 0.99 |
|  | Xie, Y. | 2015 | gastric cancer | China | Asian | HB | 7 | 86 | 45 | 6 | 35 | 71 | 36 |  | 0.99 |
|  | Zhao, Y. | 2015 | colorectal cancer | China | Asian | HB | 7 | 113 | 45 | 5 | 107 | 33 | 2 |  | 0.76 |
|  | Martin-Guerrero, I. | 2015 | Lymphocytic Leukemia | Spanish | Caucasian | HB | 7 | 48 | 41 | 10 | 138 | 164 | 40 | Taqman | 0.41 |
|  | Ye, Y. | 2008 | esophageal cancer | American | Caucasian | HB | 7 | 127 | 139 | 38 | 166 | 115 | 20 | SNPlex | 0.99 |
|  | Matthew F.Buas | 2015 | esophageal cancer | - | Caucasian | HB | 7 | 2760 | 2470 | 553 | 1525 | 1370 | 307 |  | 0.98 |

**Table S1 (continued)**

| **gene** | **reference** | **Year** | **Cancer type** | **Country** | **Ethnicity** | **controls** | **nos** | **Genotype-case** | | | **Genotype-control** | | | **method** | **HWE P-value** |
| --- | --- | --- | --- | --- | --- | --- | --- | --- | --- | --- | --- | --- | --- | --- | --- |
| ***RAH*** | rs3803012A>G |  |  |  |  |  |  | **AA** | **AG** | **GG** | **AA** | **AG** | **GG** |  |  |
|  | Chen, J. | 2013 | cervical carcinoma | China | Asian | HB | 7 | 1325 | 141 | 5 | 1397 | 129 | 3 | TaqMan | 0.99 |
|  | Jiang, Y | 2013 | breast cancer | China | Asian | HB | 7 | 766 | 92 | 12 | 772 | 107 | 5 | TaqMan | 0.54 |
|  | Liu, L. | 2013 | hepatocellular carcinoma | China | Asian | HB | 7 | 1158 | 95 | 3 | 1241 | 98 | 1 | TaqMan | 0.51 |
|  | Ma, H. | 2012 | head and neck cancer | China | Asian | HB | 7 | 344 | 45 | 2 | 799 | 91 | 2 | TaqMan | 0.73 |
|  | Zhang Qin | 2012 | Gastric cancer | China | Asian | HB | 7 | 1517 | 133 | 4 | 1674 | 168 | 2 | TaqMan | 0.29 |
| ***DICER*** | rs1057035T>C |  |  |  |  |  |  | **TT** | **TC** | **CC** | **TT** | **TC** | **CC** |  |  |
|  | Ma, H. | 2012 | head and neck cancer | China | Asian | HB | 7 | 317 | 75 | 2 | 678 | 204 | 9 | TaqMan | 0.14 |
|  | Chen, J. | 2013 | cervical carcinoma | China | Asian | HB | 7 | 1163 | 293 | 19 | 1198 | 307 | 23 | TaqMan | 0.51 |
|  | Liu, L. | 2013 | hepatocellular carcinoma | China | Asian | HB | 7 | 1063 | 196 | 16 | 1044 | 278 | 17 | TaqMan | 0.76 |
|  | Slaby, O. | 2013 | sporadic colorectal cancer | Czech | Caucasian | HB | 7 | 96 | 82 | 20 | 80 | 91 | 32 | TLDA | 0.47 |
|  | Yuan, L. | 2013 | bladder cancer | China | Asian | HB | 7 | 548 | 120 | 17 | 577 | 145 | 8 | TaqMan | 0.74 |
|  | Jiang, Y | 2013 | breast cancer | China | Asian | HB | 7 | 696 | 145 | 7 | 711 | 161 | 9 | TaqMan | 0.97 |
|  | Sung, H. | 2011 | breast cancer | Korea | Asian | HB | 7 | 463 | 92 | 5 | 459 | 102 | 6 | TaqMan | 0.90 |
|  | Zu, Y. | 2013 | lung cancer | China | Asian | HB | 7 | 380 | 190 | 30 | 350 | 210 | 40 | TaqMan | 0.27 |
|  | Martin-Guerrero, I. | 2015 | Lymphocytic Leukemia | Spanish | Caucasian | HB | 7 | 46 | 32 | 26 | 156 | 152 | 38 | Taqman | 0.91 |
|  | Zhang Qin | 2012 | gastric cancer | China | Asian | HB | 7 | 1336 | 282 | 26 | 1500 | 325 | 15 | Taqman | 0.57 |

**Table S1 (continued)**

| **gene** | **reference** | **Year** | **Cancer type** | **Country** | **Ethnicity** | **controls** | **nos** | **Genotype-case** | | | **Genotype-control** | | | **method** | **HWE *P*-value** |
| --- | --- | --- | --- | --- | --- | --- | --- | --- | --- | --- | --- | --- | --- | --- | --- |
| ***DICER*** | rs3742330A>G |  |  |  |  |  |  | **AA** | **AG** | **GG** | **AA** | **AG** | **GG** |  |  |
|  | Kim, J. S. | 2010 | lung cancer | Korea | Asian | HB | 7 | 31 | 45 | 24 | 41 | 40 | 19 |  | 0.11 |
|  | Xie, Y. | 2015 | gastric cancer | China | Asian | HB | 7 | 75 | 53 | 9 | 54 | 67 | 21 |  | 0.98 |
|  | Yuan, L. | 2013 | bladder cancer | China | Asian | HB | 7 | 548 | 120 | 17 | 577 | 145 | 8 | TaqMan | 0.74 |
|  | Zhao, Y. | 2015 | colorectal cancer | China | Asian | HB | 7 | 92 | 61 | 10 | 54 | 67 | 21 |  | 0.98 |
|  | Zheng L | 2013 | esophageal cancer | China | Asian | HB | 7 | 129 | 196 | 55 | 144 | 183 | 51 |  | 0.55 |
|  | Horikawa, Y. | 2008 | renal cell carcinoma | American | Caucasian | PB | 8 | 238 | 38 | 1 | 233 | 44 | 1 | SNPlex | 0.48 |
|  | Ye, Y. | 2008 | esophageal cancer | American | Caucasian | HB | 7 | 280 | 62 | 3 | 293 | 51 | 2 | SNPlex | 0.89 |
|  | Cho, S. H. | 2015 | Colorectal Cancer | Korean | Asian | HB | 7 | 125 | 207 | 76 | 145 | 181 | 74 | PCR-RFLP | 0.19 |
|  | Yang, H. | 2008 | bladder cancer | American | Caucasian | HB | 7 | 594 | 126 | 7 | 606 | 113 | 5 | SNPlex | 0.91 |
| ***DICER*** | rs13078T>A |  |  |  |  |  |  | **TT** | **TA** | **AA** | **TT** | **TA** | **AA** |  |  |
|  | Kim, J. S. | 2010 | lung cancer | Korea | Asian | HB | 7 | 87 | 11 | 0 | 88 | 9 | 0 |  | 0.63 |
|  | Yuan, L. | 2013 | bladder cancer | China | Asian | HB | 7 | 603 | 75 | 1 | 640 | 78 | 5 | TaqMan | 0.13 |
|  | Jiang, Y | 2013 | breast cancer | China | Asian | HB | 7 | 785 | 75 | 4 | 809 | 81 | 2 | TaqMan | 0.99 |
|  | Horikawa, Y. | 2008 | renal cell carcinoma | American | Caucasian | PB | 8 | 176 | 91 | 10 | 190 | 80 | 8 | SNPlex | 0.90 |
|  | Yang, H. | 2008 | bladder cancer | American | Caucasian | HB | 7 | 505 | 200 | 29 | 481 | 226 | 31 | SNPlex | 0.50 |
|  | Martin-Guerrero, I. | 2015 | Lymphocytic Leukemia | Spanish | Caucasian | HB | 7 | 78 | 19 | 8 | 220 | 111 | 14 | Taqman | 1.00 |
|  | Ye, Y. | 2008 | esophageal cancer | American | Caucasian | HB | 7 | 227 | 106 | 12 | 216 | 115 | 15 | SNPlex | 0.95 |
